# Vaccination Route Determines the Kinetics and Magnitude of Nasal Innate Immune Responses in Rainbow Trout (*Oncorhynchus mykiss*)

**DOI:** 10.3390/biology9100319

**Published:** 2020-10-01

**Authors:** Fen Dong, Luca Tacchi, Zhen Xu, Scott E. LaPatra, Irene Salinas

**Affiliations:** 1 Center for Evolutionary and Theoretical Immunology, Department of Biology, University of New Mexico, Albuquerque, NM 87131, USA; fendong@unm.edu (F.D.); lucatacchy@libero.it (L.T.); 2 Department of Aquatic Animal Medicine, College of Fisheries, Huazhong Agricultural University, Wuhan 430070, China; zhenxu@mail.hzau.edu.cn; 3 Clear Springs Foods Inc., Buhl, ID 83316, USA; scott.lapatra@gmail.com

**Keywords:** nasal immunity, innate immunity, cytokines, infectious hematopoietic necrosis virus, nasal vaccines, NALT, rainbow trout

## Abstract

**Simple Summary:**

Many pathogens exploit the olfactory route to reach critical organs in the body such as the brain or lungs. Thus, effective onset of an early innate immune response in the nasal epithelium is key to stopping pathogen progression. The stimulation of nasal immunity by vaccines may depend on the type of vaccine and vaccination route. The goal of this study was to evaluate the ability of a live attenuated viral vaccine to stimulate innate immunity in the olfactory organ of rainbow trout, a teleost fish of commercial aquaculture value. The kinetics and magnitude of the innate immune response depended on the route of vaccination, with the strongest and fastest responses recorded in intranasally vaccinated fish. Injection vaccination had an intermediate effect, whereas immersion vaccination resulted in delayed and weak nasal innate immunity. Injection vaccination, even with the vehicle control, induced mortality in fingerlings, whereas nasal and immersion vaccines were safe. Challenge experiments with the live virus revealed that nasal and injected vaccines conferred very high and comparable levels of protection, but immersion vaccination only induced transient protection. In conclusion, the route of vaccination determines the type, magnitude and velocity of the innate immune response in the nasal epithelium of animals.

**Abstract:**

Many pathogens infect animal hosts via the nasal route. Thus, understanding how vaccination stimulates early nasal immune responses is critical for animal and human health. Vaccination is the most effective method to prevent disease outbreaks in farmed fish. Nasal vaccination induces strong innate and adaptive immune responses in rainbow trout and was shown to be highly effective against infectious hematopoietic necrosis (IHN). However, direct comparisons between intranasal, injection and immersion vaccination routes have not been conducted in any fish species. Moreover, whether injection or immersion routes induce nasal innate immune responses is unknown. The goal of this study is to compare the effects of three different vaccine delivery routes, including intranasal (IN), intramuscular (i.m.) injection and immersion (imm) routes on the trout nasal innate immune response. Expression analyses of 13 immune-related genes in trout nasopharynx-associated lymphoid tissue (NALT), detected significant changes in immune expression in all genes analyzed in response to the three vaccination routes. However, nasal vaccination induced the strongest and fastest changes in innate immune gene expression compared to the other two routes. Challenge experiments 7 days post-vaccination (dpv) show the highest survival rates in the IN- and imm-vaccinated groups. However, survival rates in the imm group were significantly lower than the IN- and i.m.-vaccinated groups 28 dpv. Our results confirm that nasal vaccination of rainbow trout with live attenuated IHNV is highly effective and that the protection conferred by immersion vaccination is transient. These results also demonstrate for the first time that immersion vaccines stimulate NALT immune responses in salmonids.

## 1. Introduction

Vaccination has become the most effective method of preventing infectious diseases in farmed fish [1]. The ideal vaccine must provide long-term protection at both mucosal barriers and systemic tissues. The most common vaccination strategies in farmed fish are injection (intramuscular or intraperitoneal), immersion and oral vaccination [1,2,3]. The majority of fish vaccines are delivered by injection, as it is considered the most effective vaccination route [1]. However, the stimulation of mucosal immune responses by injection vaccines may be delayed compared to mucosal vaccines [4].

Nasal immunity is key to stopping the progression of neurotropic and respiratory pathogens to other body tissues such as the lower respiratory tract or the central nervous system. Recent studies have identified nasal vaccination as an effective method to control infectious diseases in fish [5,6,7]. Nasal vaccines offer many advantages over other types of vaccines such as: (i) a needle-free delivery system; (ii) the induction of strong local and systemic immune responses; (iii) the need for low amounts of antigen. In support, nasal vaccination elicits both local and systemic innate and adaptive immune responses in rainbow trout without the need for an adjuvant [5,6]. Combined, all these aspects make nasal vaccination a very attractive mucosal vaccination route for the control of aquatic infectious diseases in farmed fish.

Immersion vaccination is one of the most desirable ways to deliver vaccines in fish farms due to the ability to mass vaccinate large numbers of fingerlings without handling them one by one. During immersion vaccination, every mucosal surface of the fish, including the olfactory organ, is exposed to the diluted vaccine for a short period of time. However, several studies have evaluated how immersion vaccination induces immune responses in the skin, gut and gills of different fish species [8,9,10], the contribution of the fish nasopharynx-associated lymphoid tissue (NALT) during the immune response of immersion vaccines is yet to be determined. Teleost NALT is formed by myeloid and lymphoid cells located at the tips and neuroepithelial regions of the olfactory lamellae that response to nasal antigens [5,11]. Previous studies using a bath infection model with the parasite *Ichthyophthirius multifiliis* (*Ich*) have revealed that this parasite infects the trout olfactory organ and that trout NALT mounts innate and adaptive immune responses against this protozoan parasite [12]. Interestingly, the same study detected the highest parasite loads in the olfactory organ compared to common target tissues such as the skin and gills 7 days after infection. These findings suggest that the olfactory organ may be a key site for antigen uptake during immersion vaccination and that NALT likely mounts immune responses to immersion vaccines.

Infectious hematopoietic necrosis virus (IHNV) is classified in the family Rhabdoviridae and causes economically important disease known as infectious hematopoietic necrosis (IHN). IHN is a problem in salmonid farms worldwide, especially for Atlantic salmon (*Salmo salar*) and rainbow trout (*Oncorhynchus mykiss*) which suffer significant morbidities and mortalities [13,14,15]. We have previously reported that nasal vaccination using a live attenuated IHNV vaccine is highly protective both 7 and 28 days post-vaccination (dpv) [5]. Interestingly, IHNV immersion vaccination only elicits moderate levels of protection in rainbow trout [16,17], but a comparison of all three routes of vaccination with the same vaccine formulation has not been conducted to date in any salmonid species.

In this study, we compared three vaccination routes (intranasal (IN), intramuscular injection (i.m.) and immersion (imm) on trout nasal innate immune responses using a live attenuated IHNV vaccine and found strong and quick immune responses in the olfactory organ IN-vaccinated group. Injection and immersion vaccines also triggered innate immune responses in rainbow trout NALT, albeit of a different magnitude and kinetics than those elicited by the IN route. Challenge experiments confirmed that immersion vaccination is not as effective against IHN compared to the nasal and injection routes. Our findings open up new questions regarding how different teleost mucosa-associated lymphoid tissues (MALT) and systemic lymphoid tissues communicate in response to pathogens and vaccines.

## 2. Materials and Methods

### 2.1. Animals

The specific-pathogen-free (spf) rainbow trout (3.8 g mean weight, Clear Springs Broodstock Operations) were obtained from Clear Springs Foods Inc. (Buhl, ID, USA). Fish were maintained in 378-L tanks that received single-pass ultraviolet-light-treated spf spring water at a constant temperature of 14.5 °C and a dissolved oxygen content of 9.2 ppm. Spf status of brood stock was confirmed routinely in the entire facility. Fish were fed twice daily with a commercial rainbow trout diet (Clear Springs Foods Inc.).

### 2.2. Vaccination Trials and Challenges

A vaccine trial was conducted with a live attenuated IHNV as described in our previous study [5]. The IHNV vaccine was experimentally generated at Clear Springs Foods by serial passage in vitro as previously described [18]. Three vaccine routes were tested: intranasal vaccine method (IN), intramuscular (i.m.) injection and immersion (imm) delivery. Groups of 600 spf rainbow trout were immunized by either pipetting 25 µL of a live attenuated IHNV suspension that contained 4 × 10^6^ plaque-forming units (PFU)/mL into the right nare (IN), or by injecting 25 µL of live attenuated IHNV suspension (4 × 10^6^ PFU/mL) into the dorsal musculature just anterior to the dorsal fin (i.m.) or by immersion (imm) in 10^5^ PFU/mL for 1 h in tank water. For IN and i.m. groups, vaccine was diluted in PBS. A mock immunized group received 25 µL of PBS both IN and i.m. and were also immersed in PBS for 1 h.

**Figure 1:**
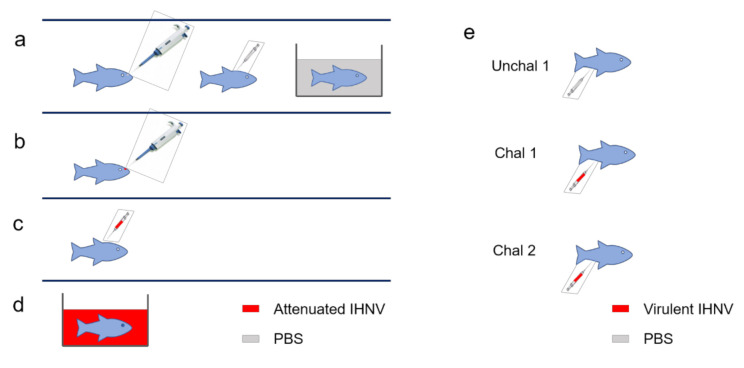
Diagram illustrating the vaccination routes and challenge experiments used in this study. (**a**) (control) received PBS into right nare, i.m. PBS injection and an 1 h immersion in PBS; (**b**) received IHNV vaccine in the right nare; (**c**) received an i.m. injection of IHNV vaccine; (**d**) received an 1 h immersion with IHNV vaccine; (**e**) Vaccination groups received PBS (Unchal 1 group, top) or virulent IHNV (Chal 1 group, (middle) and Chal 2 group (bottom) by intraperitoneal injection (i.p.) at 7 and 28 dpv, respectively. Unchal, unchallenged; Chal1 and Chal2 refer to duplicate tanks that were challenged for each experimental group at each time point.

Duplicate 25-fish groups from each treatment were challenged with IHNV at 7 and 28 dpv. Briefly, fish were anaesthetized in MS-222 (50 mg/L, Syndel, USA) and injected intraperitoneally (i.p.) with 5 (7 dpv) and 100 (28 dpv) PFU of live IHNV (isolate 220-90 [19]) (Figure 1). An additional 25-fish group from each treatment was mock-challenged by injecting PBS at each challenge interval. After virulent IHNV challenge, each 25-fish group was held in separate 19 L aquaria that received flow through 15 °C ultraviolet light-treated spring water. All groups were monitored for mortalities for 28 days post-challenge. Dead fish were monitored every day at 8 a.m. by two laboratory technicians and any dead animals removed with a clean net. Ten percent of the deceased animals were checked for presence of IHNV by plaque assay on epithelioma papulosum cyprini cells (EPC) from common carp (*Cyprinus carpio*) [20].

### 2.3. RNA Isolation and Quantitative Real-Time PCR (qPCR) Analysis

At each time point, fish (*N* = 4) were anesthetized in MS-222 and bled from the caudal vein to avoid blood contamination in the olfactory tissue. Trout olfactory organs were dissected at 1, 4 and 7 dpv (IN, i.m. or imm) and placed in sterile 1.5 mL Eppendorf tubes containing 1 mL of TRIZol (Invitrogen) and stored at −80 °C until use. Total RNA was extracted from both olfactory rosettes of each fish by homogenization using sterile tungsten carbide beads (3 mm, Qiagen) and shaking (300 times for 1 min) in a Tissuelyser II (Qiagen). RNA was extracted following a standard phenol-chloroform extraction protocol. The RNA pellet was washed in 80% ethanol, air dried and resuspended in RNase-free water. RNA concentrations were determined by spectrophotometry (Nanodrop ND1000) and the integrity of the RNA was determined by electrophoresis (Agilent Bioanalyser, 2100). RNA samples were stored at −80 ℃ until use. cDNA was synthesized using 1 μg of total RNA per sample as previously described [21].

The qPCRs reactions (25-µL reaction volume) consisted of 3 µL of a diluted cDNA template (4 ng of total RNA equivalents), 12.5 µL of Power SYBR Green PCR master mix (2×, Applied Biosystems) and 150 nM forward and reverse primers. Reactions were run in triplicate. The amplification profile consisted of an initial denaturation step at 95 ℃ for 10 min, and then 30 cycles of 95 °C for 15 s and 60 °C for 1 min followed by melting (dissociation stage) from 72 to 95 °C in an ABI Prism 7000 (Applied Biosystems) sequence detection system. A negative control (no template) reaction was also performed for each primer pair. A sample from the serial dilution was run on a 2% agarose gel and stained with Red Gel Stain and viewed under ultraviolet light to confirm a band of the correct size was amplified. In order to determine the efficiency of the amplification for each primer pair, reactions were carried out using serial tenfold dilutions of pooled cDNA on the same plate as the experimental samples. The efficiency was calculated as E = 10 ^(−1/s)^, where s is the slope generated from the serial dilutions, when Log dilution is plotted against ΔCT (threshold cycle number). Expression levels were normalized to those of the trout elongation factor 1a (*ef-1a*) which was used as a single house-keeping gene. The relative expression level of the genes was determined using the Pfaffl method [22]. The primers used for qPCR are shown in Table 1. All primer sets were designed to span an exon–exon boundary. Absence of amplification of genomic DNA contamination for each primer set was checked by standard PCR using total rainbow trout DNA samples from a pool of lymphoid tissues of vaccinated fish as template.

### 2.4. Statistical Analysis

Kaplan–Meier survival curves were plotted to state the mortality of vaccination trials and challenge experiments. Data were analyzed in Prism version 6.01 (GraphPad). Data are expressed as mean± standard error (s.e). Unpaired Student’s *t*-tests were used for analysis of differences between groups. Fisher’s exact test of conditional independence for a 2 × 2 contingency table was used for follow-up tests using Prism version 6.01. *P*-values less than 0.05 were considered statistically significant.

## 3. Results

### 3.1. Local Innate Immune Responses in Trout NALT in Response to Different Vaccination Routes

**Figure 2:**
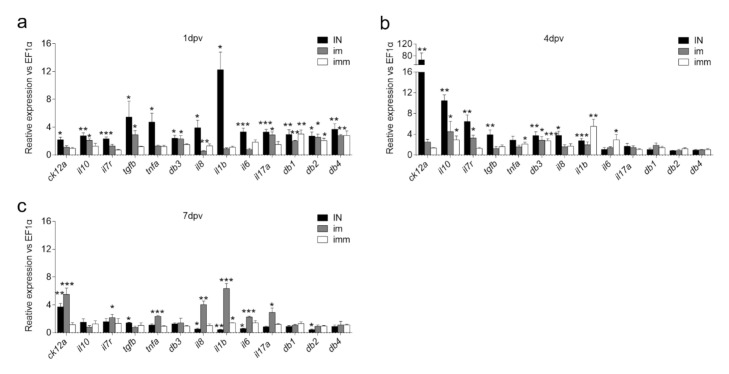
Kinetics of the innate immune response in trout NALT following nasal, injection or immersion vaccination with live attenuated IHNV vaccine. (**a**) Gene expression changes 1 dpv; (**b**) Gene expression changes 4 dpv; (**c**) Gene expression changes 7 dpv. Gene expression was quantified by quantitative real-time PCR (*N* = 4 fish per group). Data are expressed as mean fold increase in expression ± s.e. (unpaired Student’s *t*-test) *P < 0.05, ** *P* < 0.01, *** *P* < 0.001.

In this study, we vaccinated rainbow trout with live attenuated IHNV or PBS by different delivery routes (Figure 1a–d) and then investigated changes in expression of 13 immune-related genes at 1, 4 and 7 dpv in trout NALT by qPCR. These genes were selected based on a previous transcriptomics study performed in trout NALT 4 dpv [5]. At one dpv, *il1b* and *tgfb* expression were greatly up-regulated (~12- and ~5.5-fold, respectively) in the IN group, but not in the i.m. or imm groups. Moreover, the pro-inflammatory cytokines *tnfa*, *il8* and *il6* were also significantly up-regulated 1 dpv in the IN group (~4.7-, ~3.9- and ~3.3-fold) but not in the other two vaccinated groups (Figure 2a). In fact, *il8* expression was down-regulated 1.8-fold in NALT of i.m. vaccinated fish 1 dpv. The *il17a* expression was up-regulated ~3.3- and ~2.9-fold in both IN and i.m. vaccinated groups 1 dpv respectively, but not for the imm group. The expression of *ck12a*, a chief nasal chemokine in trout, was only significantly up-regulated in IN vaccinated fish 1 dpv. The expression levels of *il7r*, a marker for memory T cells following acute infections [32,33], were significantly higher (~2.3-fold) in IN vaccinated fish, with no change in expression detected in the i.m. and imm groups. Interestingly, except for the *db3* and *db4* expression in the imm-vaccinated group, all four beta defensin genes (*omdb1-4*) examined were up-regulated in trout NALT 1 dpv in all vaccinated groups compared to mock-vaccinated controls.

Four dpv, *ck12a* as the most up-regulated gene (~67-fold) in the IN group, followed by *il10*, *il7r* and *tgfb* (~10-, ~6.5- and ~4-fold, respectively) (Figure 2b). Interestingly, whereas *il1b* and *il8* expression was still significantly higher compared to controls, *il6* and *tnfa* expression levels were already similar to those of controls in the IN group. At this timepoint, expression levels of *omdb-1*, *omdb-2* and *omdb-4* had also returned to basal levels in all vaccinated groups. However, *omdb-3* expression was significantly elevated in all vaccinated groups compared to controls (~2.8–3.8-fold), indicating a unique behavior of this beta defensin in trout NALT. With regards to the i.m. and imm groups at 4 dpv, transcription levels of *tnfa*, *il1b* and *il6* were significantly higher in the imm group but not the i.m. group, indicating that pro-inflammatory responses occur in trout NALT following imm vaccination albeit with a delay (4 days vs. 1 day) compared to the IN route.

One of the most remarkable findings of this study was the difference in the transcription kinetics of the innate immune responses that occur in NALT depending on the route of vaccination. As shown in Figure 2c, i.m. injection resulted in significant up-regulation of *tnfa* (~2.4-fold), *il8* (~4-fold), *il1b* (~6.4-fold), and *il6* (~2.3-fold) expression 7 dpv, while the expression of these genes was already down-regulated in the IN group at this timepoint compared to 4 dpv and showed very modest or no change in expression in the imm group (Figure 2c). *Ck12a* expression remained significantly up-regulated in both the IN and i.m. groups (~3.7- and ~5.5-fold, respectively) but no changes in expression was recorded in the imm group (Figure 2). Regarding beta defensins, no changes in expression were observed in any of the vaccinated groups compared to controls 7 dpv except for a significant down-regulation in *omdb-2* expression (~2.3-fold) in the IN vaccinated group (Figure 2c,d).

Taken together, these data show that IN vaccination triggers quick (day 1) and potent pro-inflammatory and anti-inflammatory immune responses in trout NALT and that these responses are rapidly dampened by day 7. Imm vaccination also results in innate immune responses in trout NALT but these have lower magnitude and a delayed onset (day 4) compared to those elicited by IN vaccination. Finally, i.m. injection vaccination also induces innate immune responses in trout NALT, but those occurred even later (day 7) compared to the other two vaccination routes and with a magnitude more similar to the imm group than to the IN group.

### 3.2. Protection Against IHNV Challenge

**Figure 3:**
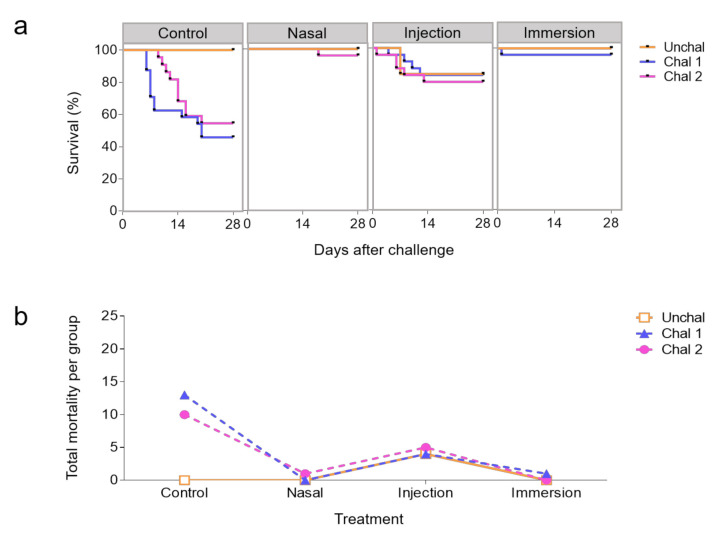
Protection conferred by live attenuated IHNV vaccine in trout. Survival curves (**a**) and total mortality (**b**) of trout vaccinated with live attenuated IHNV intranasally (IN), by intramuscular (i.m.) injection or by immersion (imm). Trout (*N* = 25 per tank in duplicate tanks) were challenged to virulent IHNV i.p. 7 dpv (5 PFU). Unchal, unchallenged; Chal1 and Chal2 refer to duplicate tanks that were challenged for each experimental group.

Percent cumulative mortalities for all vaccination trials are shown in Table 2. Fisher’s exact tests show the survival in different vaccinated groups and challenged to pathogen (Table 3). At 7dpv, survival rates in the IN and imm groups ranged between 96% and 100% (*P*-value = 1 IN vs. i.m., Table 3), whereas the unvaccinated group had a mean survival rate of 50% (Figure 3a). I.m. vaccinated and challenged fish had a mean survival rate of 81.3% (Figure 3a). I.m. vaccination without challenge resulted in a survival rate of 84% (percent cumulative mortality = 16%) (Figure 3b and Table 2, Fisher’s exact test *P-*value = 0.1099), indicating losses due to the injection procedure and not the pathogen challenge in some fingerlings.

**Figure 4:**
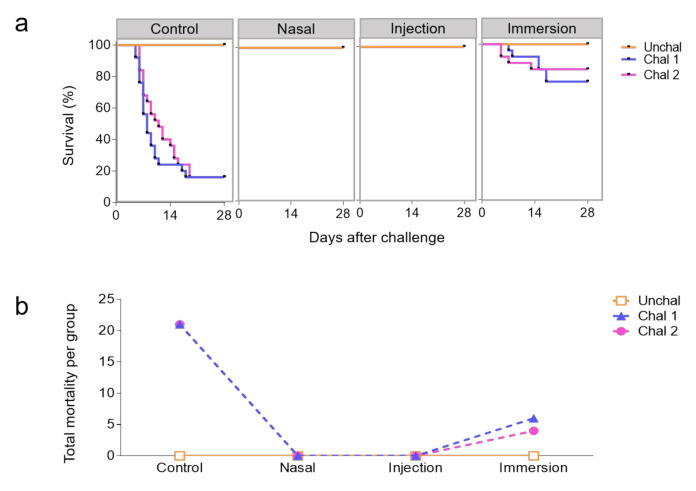
Protection conferred by live attenuated IHNV vaccine in trout. Survival curves (**a**) and total mortality (**b**) of trout vaccinated with live attenuated IHNV intranasally (IN), by intramuscular (i.m.) injection or by immersion (imm). Trout (*N* = 25 per tank in duplicate tanks) were challenged to virulent IHNV i.p. 28 dpv (100 PFU). Unchal, unchallenged; Chal1 and Chal2 refer to duplicate tanks that were challenged for each experimental group.

At 28 dpv, the mean survival rate of the unvaccinated and challenged group was 84%. The mean survival rate of the challenged imm group was 80% compared to the IN and i.m. groups, which showed 100% protection (Figure 4a,b). Fisher’s exact tests show sufficient evidence for differences between the imm vaccinated group and the IN- or i.m.-vaccinated group (*P*-value = 0.0012 and 0.0018, respectively, Table 3) and no differences between the IN and i.m. groups (*P*-value = 1, Table 3).

## 4. Discussion

Vaccination has become the most effective method of preventing infectious diseases in farmed fish. The majority of the fish vaccines are delivered by injection, as it is still considered the most effective vaccination route [1]. Injection vaccination, however, is labor-intensive and can result in side-effects that impact fish welfare [1]. Injected vaccines do not directly stimulate mucosal surfaces, the first tissues to be infected by pathogens. Mucosal vaccines for fish, such as immersion or oral vaccination may be the simplest and most cost-effective vaccination methods, especially is small fish, but they usually result in sub-optimal and short-term protection [1,34]. To date, very few viral vaccines have been developed for immersion because of their low efficacy [35]. Nasal vaccination, although labor-intensive, has been shown to be effective against viral and bacterial diseases, to be safe, and to stimulate both mucosal and systemic immunity in fish [5,7]. Importantly, nasal vaccination provides additional welfare benefits for the fish as well as the handling staff, since needle use is avoided.

Vaccines based on live attenuated viruses have been amongst the most successful, cost-effective immune interventions in medical history [36,37]. Live attenuated viral vaccines for fish typically elicit a strong and sustained immune response to the target disease [38]. Attenuated live vaccines are safe under most circumstances, although there are some risks such as the presence of residual virulence in vaccinates or virulence in immunocompromised hosts [3]. In our results, we found no mortality in the IN-vaccinated group after challenge with virulent IHNV, demonstrating once again that nasal delivery in rainbow trout is safe. Furthermore, challenge experiments showed that the nasal route is very effective both at 7 and 28 dpv. Some of these benefits are illustrated in the data from the present study, where we recorded 16% mortality in the unchallenged i.m.-vaccinated group but no mortalities in the unchallenged IN or imm groups. Although immersion vaccination is less labor-intensive and mimics natural exposure to infection [35], our data confirm that immersion vaccination with a live viral vaccine only affords short-lived immunity in rainbow trout, even in a prolonged exposure set-up like the one used in this study.

Teleost NALT shares the main features of other teleost mucosa-associated lymphoid tissues (MALT) and mounts strong immune responses following infection or vaccination [5,12]. Importantly, previous studies in trout demonstrated that intranasal antigen delivery results in very rapid and potent innate immune responses [5,6] and modest circulating specific antibody titers [39]. Here, we evaluated the expression of 13 immune-related genes including cytokines, *il7r*, the chemokine *ck12a* and four β-defensins and found unique kinetics in the local NALT immune responses depending on the route of immunization.

Neuronal tissues such as the olfactory epithelium are particularly susceptible to pathological inflammation and, therefore, tight regulation of pro-inflammatory immune responses is critical [40]. Our gene expression studies clearly show a rapid pro-inflammatory state in trout NALT following nasal IHNV vaccination. The pro-inflammatory response was characterized by the elevated expression of classical cytokines including *il1b*, *il6*, *il8* and *tnfa*. Interestingly, our time series study revealed that the pro-inflammatory signature was no longer detected by 7 dpv. Moreover, elevated expression of anti-inflammatory cytokines *il10* and *tgfb* was also recorded early on in response to intranasal IHNV vaccination, suggesting a tissue repair response. Combined, these results highlight the ability of NALT to mount inflammatory responses against novirhabdoviruses while limiting the magnitude and duration of this response in order to protect tissue integrity.

IL-7 and its receptor IL-7R play critical roles in B and T cell growth, maturation and activation [41,42]. Additionally, IL-7 is involved in CD4^+^ and CD8^+^ memory T cell responses [43]. At mucosal barriers, IL-7 promotes IL-17A responses against respiratory bacterial infection [44] and aids in the elimination of activated lymphocytes in the inflamed mucosa [45]. Our data show that IN and i.m. vaccination induce *il7r* expression in NALT, however, immersion vaccination results in transient down-regulation or no changes in NALT *il7r* expression. This important finding may reflect the need to regulate inflammatory lymphocytes in the olfactory tissue in response to high antigenic doses such as those applied in nasal vaccination with this vaccine. Moreover, the differential regulation of *il7r* expression noted in each experimental group may shape the local and systemic B and T cell responses elicited by each of the vaccination routes. Further studies are needed to support or reject this hypothesis.

The β-defensin family of genes plays a significant role in antibacterial and antiviral immunity in fish [27,46,47,48]. Previous studies showed up-regulation of β-defensins and other antimicrobial peptide (AMP) genes in the kidney of brown trout infected with the novirhabodvirus viral hemorrhagic septicemia virus (VHSV) [49]. However, the expression of antimicrobial peptides in the trout olfactory organ had not been investigated to date. Our results highlight the key role of β-defensins as early antimicrobial effectors, in this case, in response to a viral antigen. Importantly, induction of β-defensin gene expression in NALT was highest when the vaccine was delivered intranasally, compared to the other two routes. We observed that one of the four β-defensin genes studied, *omdb-3*, showed a unique expression pattern characterized by a more sustained up-regulation compared to the other three β-defensin genes. Similarly, in VHSV-infected brown trout, *omdb-3* expression changes in the kidney differed from those of other AMP genes [49]. Future studies should determine the unique function of each AMP in the context of nasal immunity.

Chemokines play pivotal roles in coordinating leukocyte migration in immunity and inflammation [50,51]. In rainbow trout, the CCL19-like chemokine CK12 is strongly expressed both at the mRNA and protein level in mucosal tissues such as gill, gut and skin, suggesting its role as a mucosal chemokine [52]. Local nasal production of CK12a plays a central role in antiviral immune protection both locally and systemically in trout [23]. Specifically, CK12a is chemotactic in vitro and in vivo and recruits CD8α^+^ lymphocytes to the nasal mucosa [23]. In line with these findings, our results show that the *ck12a* mRNA levels increase significantly in NALT after immunization with live attenuated IHNV vaccine both by nasal delivery and injection. Previous studies revealed increased *ck12* expression in the liver of trout infected by i.p. injection with VHSV [53] and in the fin bases of trout following bath infection with VHSV [54]. Remarkably, we found that the immersion vaccinated group showed no change in *ck12a* expression in NALT, suggesting that this route does not effectively stimulate this chemokine in the olfactory organ. Therefore, it is unlikely that recruitment of immune cells such as CD8α^+^ lymphocytes into the olfactory organ occurs in response to this vaccination mode, perhaps explaining the lower effectiveness of this route compared to the other two. Although we did not evaluate *ck12a* responses at other MALT in our immersion experiment, such studies will reveal if our observation in trout NALT is unique to NALT or universal across all MALT following immersion vaccination.

One of the caveats of the present study is that we did not measure transcriptional type I IFN responses or IFN-stimulated genes (ISGs), known to play an important role in teleost antiviral immunity [55,56,57]. We previously reported strong modulation of antiviral immunity genes in rainbow trout NALT 4 days post nasal vaccination with live attenuated IHNV [5]. Given the rapid onset of type I IFN responses against novirhabdoviruses previously found in other studies in teleosts [58,59], we predict that, similar to the genes investigated here, the vaccination route and antigen dose will be major determining factors of the type I IFN response in trout NALT.

The teleost olfactory organ, although not a respiratory surface, shares many anatomical, cellular and molecular features with the mammalian olfactory system [60]. Thus, teleost fish models have been proposed as comparative models for human nasal infections and immunity [61]. Several viral pathogens infect the human host via the nasal epithelium including influenza virus [62] and SARS-CoV-2 [63]. Understanding innate immunity in the nasal mucosa is therefore critical for the effective design of immunoprophylactic strategies against respiratory and neurotropic viruses. Interestingly, currently, none of the COVID-19 vaccines currently being tested in clinical trials are being delivered intranasally. Our results indicate the immediate and potent immune responses in the trout nasal mucosa are best achieved when vaccines are delivered intranasally, and it is likely that this is the case in humans too.

In conclusion, the present study shows that trout NALT mounts innate immune responses after vaccination with live attenuated IHNV vaccine regardless of the route of vaccination. Importantly, the route of vaccination and antigen dose determine the magnitude, type and kinetics of the NALT innate immune responses. Specifically, the responses induced by direct delivery of the vaccine into the nasal cavity of trout are not mimicked by neither injection nor immersion vaccination. Finally, future studies should further investigate whether the limited stimulation of mucosal immune responses in NALT by immersion vaccination may explain the suboptimal protection conferred by this route in this or other vaccine models.

## 5. Conclusions

This study shows that nasal vaccination with a live attenuated IHNV vaccine induces fast and potent innate immune responses in trout NALT. Intramuscular vaccination with live attenuated IHNV vaccine induces a slightly delayed and less potent innate immune responses in trout NALT. Immersion vaccination with live attenuated IHNV vaccine causes delayed and mild innate immune responses in trout NALT. Finally, whilst nasal and injection vaccination are highly protective against IHN, immersion vaccination provides only transient protection.

## Figures and Tables

**Table 1:** Primers used in this study.

Gene	Primer Name	Primer Sequence (5′-3′)	Amplicon Size	Reference
*ck12*	*ck12a* F	CTCTGAGGTACCCGTGGATTGC	277 bp	[22]
	*ck12a* R	CCTTAGGGACTATTGTTCTTCAGC		
*il10*	*il10* F	CTGCTGGACGAAGGGATTCTAC	277 bp	[23]
	*il10* R	GGCCTTTATCCTGCATCTTCTC		
*il7r*	*il7r* F	GTGGAGAAGAATTGGTTGAC	117 bp	[24]
	*il7r* R	CCTCCATTTCATCATCGGTGTC		
*tgfb*	*tgfb* F	CATGTCCATCCCCCAGAACT	361 bp	[25]
	*tgfb* R	GGACAACTGTTCCACCTTGTGTT		
*tnfa*	*tnfa* F	GGGGACAAACTGTGGACTGA	66 bp	[26]
	*tnfa* R	GAAGTTCTTGCCCTGCTCTG		
*omdb-3*	*omdb-3* F	GCTTGTGGAATACAAGAGTCATCTGC	138 bp	[27]
	*omdb-3* R	GCATACATTCGGCCATGTACATCC		
*il8*	*il8* F	AGAATGTCAGCCAGCCTTGT	69 bp	[28]
	*il8* R	TCTCAGACTCATCCCCTCAGT		
*il1b*	*il1b* F	ACATTGCCAACCTCATCATCG	91 bp	[29]
	*il1b* R	TTGAGCAGGTCCTTGTCCTTG		
*il6*	*il6* F	ACTCCCCTCTGTCACACACC	295 bp	[26]
	*il6* R	GGCAGACAGGTCCTCCACTA		
*il17a*	*il17a* F	CGTGTCGAAGTACCTGGTTGTGT	212 bp	[30]
	*il17a* R	GGTTCTCCACTGTAGTGCTTTTCCA		
*omdb-1*	*omdb-1* F	GGTTTTCCTATTGCTTAATGTTGTGG	302 bp	[27]
	*omdb-1* R	GACACACAGTTAAGTCATGG		
*omdb-2*	*omdb-2* F	GCTGACAGCAGTGCAAGCTGATGACAC	143 bp	[27]
	*omdb-2* R	GCAAAGCACAGCATCTTAATCTGC		
*omdb-4*	*omdb-4* F	GCAACTCTTCTAAAGAACAGT	238 bp	[27]
	*omdb-4* R	CGTGGGCGACACAGCATACAAATCC		
*ef-1a*	*ef-1a* F	CAAGGATATCCGTCGTGGCA	353 bp	[31]
	*ef-1a* R	ACAGCGAAACGACCAAGAGG		

**Table 2:** Percent (%) cumulative mortality for each treatment group.

	7 Dpv			28 Dpv		
	Unchal	Chal 1	Chal 2	Unchal	Chal 1	Chal 2
control	0	54.2	45.5	0	84	84
IN	0	0	4	0	0	0
i.m.	16	16.7	20.8	0	0	0
imm	0	4	0	0	24	16

**Table 3:** Statistical analyses of the survival between the different vaccinated groups and challenged to pathogen. Significance symbols indicate the *P*-value: * *P* < 0.05, ** *P* < 0.01, *** *P* < 0.001.

Dpv	Pathogen	Vaccinated	*P-*Value	Significance
7	IHNV	control vs. IN	<0.0001	***
7	IHNV	control vs. i.m.	0.0021	**
7	IHNV	control vs. imm	<0.0001	***
7	IHNV	IN vs. i.m.	0.0072	**
7	IHNV	IN vs. imm	1	
7	IHNV	i.m. vs. imm	0.0072	**
7	none	control vs. IN	1	
7	none	control vs. i.m.	0.1099	
7	none	control vs. imm	1	
7	none	IN vs. i.m.	0.1099	
7	none	IN vs. imm	1	
7	none	i.m. vs. imm	0.1099	
28	IHNV	control vs. IN	<0.0001	***
28	IHNV	control vs. i.m.	<0.0001	***
28	IHNV	control vs. imm	<0.0001	***
28	IHNV	IN vs. i.m.	1	
28	IHNV	IN vs. imm	0.0012	**
28	IHNV	i.m. vs. imm	0.0018	**
28	none	control vs. IN	1	
28	none	control vs. i.m.	1	
28	none	control vs. imm	1	
28	none	IN vs. i.m.	1	
28	none	IN vs. imm	1	
28	none	i.m. vs. imm	1	

## References

[1] Plant K.P., Lapatra S.E. (2011). Advances in fish vaccine delivery. Dev. Comp. Immunol..

[2] Brudeseth B.E., Wiulsrød R., Fredriksen B.N., Lindmo K., Løkling K.E., Bordevik M., Steine N., Klevan A., Gravningen K. (2013). Status and future perspectives of vaccines for industrialised fin-fish farming. Fish Shellfish Immunol..

[3] Ma J., Bruce T.J., Jones E.M., Cain K.D. (2019). A Review of Fish Vaccine Development Strategies: Conventional Methods and Modern Biotechnological Approaches. Microorganisms.

[4] Zhang H., Shen B., Wu H., Gao L., Liu Q., Wang Q., Xiao J., Zhang Y. (2014). Th17-like immune response in fish mucosal tissues after administration of live attenuated *Vibrio anguillarum* via different vaccination routes. Fish Shellfish Immunol..

[5] Tacchi L., Musharrafieh R., Larragoite E.T., Crossey K., Erhardt E.B., Martin S.A.M., LaPatra S.E., Salinas I. (2014). Nasal immunity is an ancient arm of the mucosal immune system of vertebrates. Nat. Commun..

[6] LaPatra S., Kao S., Erhardt E.B., Salinas I. (2015). Evaluation of dual nasal delivery of infectious hematopoietic necrosis virus and enteric red mouth vaccines in rainbow trout (*Oncorhynchus mykiss*). Vaccine.

[7] Salinas I., LaPatra S.E., Erhardt E.B. (2015). Nasal vaccination of young rainbow trout (*Oncorhynchus mykiss*) against infectious hematopoietic necrosis and enteric red mouth disease. Dev. Comp. Immunol..

[8] Esteve-Gassent M.D., Fouz B., Amaro C. (2004). Efficacy of a bivalent vaccine against eel diseases caused by *Vibrio vulnificus* after its administration by four different routes. Fish Shellfish Immunol..

[9] Sheng X., Chai B., Wang Z., Tang X., Xing J., Zhan W. (2019). Polymeric immunoglobulin receptor and mucosal IgM responses elicited by immersion and injection vaccination with inactivated Vibrio anguillarum in flounder (*Paralichthys olivaceus*). Aquaculture.

[10] Hoare R., Ngo T.P.H., Bartie K.L., Adams A. (2017). Efficacy of a polyvalent immersion vaccine against *Flavobacterium psychrophilum* and evaluation of immune response to vaccination in rainbow trout fry (Onchorynchus mykiss L.). Vet. Res..

[11] Sepahi A., Casadei E., Tacchi L., Muñoz P., LaPatra S.E., Salinas I. (2016). Tissue microenvironments in the nasal epithelium of rainbow trout (*Oncorhynchus mykiss*) define two distinct CD8α^+^ cell populations and establish regional immunity. J. Immunol..

[12] Yu Y.Y., Kong W., Yin Y.X., Dong F., Huang Z.Y., Yin G.M., Dong S., Salinas I., Zhang Y.A., Xu Z. (2018). Mucosal immunoglobulins protect the olfactory organ of teleost fish against parasitic infection. PLoS Pathog..

[13] Dixon P., Paley R., Alegria-Moran R., Oidtmann B. (2016). Epidemiological characteristics of infectious hematopoietic necrosis virus (IHNV): A review. Vet. Res..

[14] Ammayappan A., LaPatra S.E., Vakharia V.N. (2010). Molecular characterization of the virulent infectious hematopoietic necrosis virus (IHNV) strain 220-90. Virol. J..

[15] Bootland L.M., Leong J.C., Woo P.T.K., Bruno D.W. (1999). Infectious Hematopoietic Necrosis Virus.

[16] Noonan B., Enzmann P.J., Trust T.J. (1995). Recombinant infectious hematopoietic necrosis virus and viral hemorrhagic septicemia virus glycoprotein epitopes expressed in Aeromonas salmonicida induce protective immunity in rainbow trout (*Oncorhynchus mykiss*). Appl. Environ. Microbiol..

[17] Yong C.Y., Ong H.K., Tang H.C., Yeap S.K., Omar A.R., Ho K.L., Tan W.S. (2019). Infectious hematopoietic necrosis virus: Advances in diagnosis and vaccine development. PeerJ.

[18] LaPatra S., Clouthier S., Anderson E. (2004). Current Trends in Immunotherapy and Vaccine Development for Viral Diseases of Fish. Current Trends in the Study of Bacterial and Viral Fish and Shrimp Diseases.

[19] LaPatra S.E., Lauda K.A., Jones G.R., Walker S.C., Shewmaker W.D. (1994). Development of passive immunotherapy for control of infectious hematopoietic necrosis. Dis. Aquat. Org..

[20] LaPatra S.E., Roberti K.A., Rohovec J.S., Fryer J.L. (1989). Fluorescent Antibody Test for the Rapid Diagnosis of Infectious Hematopoietic Necrosis. J. Aquat. Anim. Health.

[21] Tacchi L., Larragoite E., Salinas I. (2013). Discovery of J chain in African lungfish (*Protopterus dolloi*, Sarcopterygii) using high throughput transcriptome sequencing: Implications in mucosal immunity. PLoS ONE.

[22] Pfaffl M.W. (2001). A new mathematical model for relative quantification in real-time RT-PCR. Nucleic Acids Res..

[23] Sepahi A., Tacchi L., Casadei E., Takizawa F., LaPatra S.E., Salinas I. (2017). CK12a, a CCL19-like chemokine that orchestrates both nasal and systemic antiviral immune responses in rainbow trout. J. Immunol..

[24] Costa M.M., Maehr T., Diaz-Rosales P., Secombes C.J., Wang T. (2011). Bioactivity studies of rainbow trout (*Oncorhynchus mykiss*) interleukin-6: Effects on macrophage growth and antimicrobial peptide gene expression. Mol. Immunol..

[25] Maehr T., Costa M.M., González Vecino J.L., Wadsworth S., Martin S.A.M., Wang T., Secombes C.J. (2013). Transforming growth factor-β1b: A second TGF-β1 paralogue in the rainbow trout (*Oncorhynchus mykiss*) that has a lower constitutive expression but is more responsive to immune stimulation. Fish Shellfish Immunol..

[26] Jørgensen T.R., Raida M.K., Kania P.W., Buchmann K. (2009). Response of rainbow trout (*Oncorhynchus mykiss*) in skin and fin tissue during infection with a variant of *Gyrodactylus salaris* (Monogenea: Gyrodactylidae). Folia Parasitol..

[27] Casadei E., Wang T., Zou J., González Vecino J.L., Wadsworth S., Secombes C.J. (2009). Characterization of three novel β-defensin antimicrobial peptides in rainbow trout (*Oncorhynchus mykiss*). Mol. Immunol..

[28] Raida M.K., Buchmann K. (2008). Bath vaccination of rainbow trout (*Oncorhynchus mykiss* Walbaum) against *Yersinia ruckeri*: Effects of temperature on protection and gene expression. Vaccine.

[29] Zou J., Grabowski P.S., Cunningham C., Secombes C.J. (1999). Molecular cloning of interleukin 1beta from rainbow trout Oncorhynchus mykiss reveals no evidence of an ice cut site. Cytokine.

[30] Harun N.O., Wang T., Secombes C.J. (2011). Gene expression profiling in naïve and vaccinated rainbow trout after Yersinia ruckeri infection: Insights into the mechanisms of protection seen in vaccinated fish. Vaccine.

[31] Díaz-Rosales P., Bird S., Wang T.H., Fujiki K., Davidson W.S., Zou J., Secombes C.J. (2009). Rainbow trout interleukin-2: Cloning, expression and bioactivity analysis. Fish Shellfish Immunol..

[32] Huster K.M., Busch V., Schiemann M., Linkemann K., Kerksiek K.M., Wagner H., Busch D.H. (2004). Selective expression of IL-7 receptor on memory T cells identifies early CD40L-dependent generation of distinct CD8+ memory T cell subsets. Proc. Natl. Acad. Sci. USA.

[33] Kaech S.M., Tan J.T., Wherry E.J., Konieczny B.T., Surh C.D., Ahmed R. (2003). Selective expression of the interleukin 7 receptor identifies effector CD8 T cells that give rise to long-lived memory cells. Nat. Immunol..

[34] Munang’andu H.M., Mutoloki S., Evensen Ø. (2015). An overview of challenges limiting the design of protective mucosal vaccines for finfish. Front. Immunol..

[35] Bøgwald J., Dalmo R.A. (2019). Review on immersion vaccines for fish: An update 2019. Microorganisms.

[36] Minor P.D. (2015). Live attenuated vaccines: Historical successes and current challenges. Virology.

[37] Lauring A.S., Jones J.O., Andino R. (2010). Rationalizing the development of live attenuated virus vaccines. Nat. Biotechnol..

[38] Dhar A.K., Manna S.K., Thomas Allnutt F.C. (2014). Viral vaccines for farmed finfish. Virus Dis..

[39] Magadan S., Jouneau L., Boudinot P., Salinas I. (2019). Nasal vaccination drives modifications of nasal and systemic antibody repertoires in rainbow trout. J. Immunol..

[40] Sepahi A., Kraus A., Casadei E., Johnston C.A., Galindo-Villegas J., Kelly C., García-Moreno D., Muñoz P., Mulero V., Huertas M. (2019). Olfactory sensory neurons mediate ultrarapid antiviral immune responses in a TrkA-dependent manner. Proc. Natl. Acad. Sci. USA.

[41] Corfe S.A., Paige C.J. (2012). The many roles of IL-7 in B cell development; mediator of survival, proliferation and differentiation. Semin. Immunol..

[42] Niu N., Qin X. (2013). New insights into IL-7 signaling pathways during early and late T cell development. Cell Mol. Immunol..

[43] Kieper W.C., Tan J.T., Bondi-Boyd B., Gapin L., Sprent J., Ceredig R., Surh C.D. (2002). Overexpression of interleukin (IL)-7 leads to IL-15-independent generation of memory phenotype CD8^+^ T cells. J. Exp. Med..

[44] Hassane M., Jouan Y., Creusat F., Soulard D., Boisseau C., Gonzalez L., Patin E.C., Heuzé-Vourc’h N., Sirard J.C., Faveeuw C. (2020). Interleukin-7 protects against bacterial respiratory infection by promoting IL-17A-producing innate T-cell response. Mucosal Immunol..

[45] Watanabe M., Ueno Y., Yamazaki M., Hibi T. (1999). Mucosal IL-7-mediated immune responses in chronic colitis-IL-7 transgenic mouse model. Immunol. Res..

[46] Zhou Y., Lei Y., Cao Z., Chen X., Sun Y., Xu Y., Guo W., Wang S., Liu C. (2019). A β-defensin gene of Trachinotus ovatus might be involved in the antimicrobial and antiviral immune response. Dev. Comp. Immunol..

[47] Dong J.J., Wu F., Ye X., Sun C.F., Tian Y.Y., Lu M.X., Zhang R., Chen Z.H. (2015). Β-defensin in Nile tilapia (*Oreochromis niloticus*): Sequence, tissue expression, and anti-bacterial activity of synthetic peptides. Gene.

[48] Zhu J., Wang H., Wang J., Wang X., Peng S., Geng Y., Wang K., Ouyang P., Li Z., Huang X. (2017). Identification and characterization of a β-defensin gene involved in the immune defense response of channel catfish, Ictalurus punctatus. Mol. Immunol..

[49] Gorgoglione B., Taylor N.G.H., Holland J.W., Feist S.W., Secombes C.J. (2019). Immune response modulation upon sequential heterogeneous co-infection with *Tetracapsuloides bryosalmonae* and VHSV in brown trout (Salmo trutta). Fish Shellfish Immunol..

[50] Legler D.F., Thelen M. (2016). Chemokines: Chemistry, biochemistry and biological function. Chim. Int. J. Chem..

[51] Viola A., Luster A.D. (2008). Chemokines and their receptors: Drug targets in immunity and inflammation. Annu. Rev. Pharmacol. Toxicol..

[52] Montero J., Ordas M.C., Alejo A., Gonzalez-Torres L., Sevilla N., Tafalla C. (2011). CK12, a rainbow trout chemokine with lymphocyte chemo-attractant capacity associated to mucosal tissues. Mol. Immunol..

[53] Castro R., Abós B., Pignatelli J., von Gersdorff Jørgensen L., González Granja A., Buchmann K., Tafalla C. (2014). Early immune responses in rainbow trout liver upon viral hemorrhagic septicemia virus (VHSV) infection. PLoS ONE.

[54] Montero J., Garcia J., Ordas M.C., Casanova I., Gonzalez A., Villena A., Coll J., Tafalla C. (2011). Specific regulation of the chemokine response to viral hemorrhagic septicemia virus at the entry site. J. Virol..

[55] Boudinot P., Langevin C., Secombes C.J., Levraud J.-P. (2016). The peculiar characteristics of fish type I interferons. Viruses.

[56] Langevin C., Aleksejeva E., Passoni G., Palha N., Levraud J.-P., Boudinot P. (2013). The Antiviral Innate Immune Response in Fish: Evolution and Conservation of the IFN System. J. Mol. Biol..

[57] Rao Y., Su J. (2015). Insights into the antiviral immunity against grass carp (Ctenopharyngodon idella) reovirus (GCRV) in grass carp. J. Immunol. Res..

[58] Ballesteros N.A., Alonso M., Saint-Jean S.R., Perez-Prieto S.I. (2015). An oral DNA vaccine against infectious haematopoietic necrosis virus (IHNV) encapsulated in alginate microspheres induces dose-dependent immune responses and significant protection in rainbow trout (Oncorrhynchus mykiss). Fish Shellfish Immunol..

[59] Nombela I., Carrion A., Puente-Marin S., Chico V., Mercado L., Perez L., Coll J., Ortega-Villaizan M.D.M. (2017). Infectious pancreatic necrosis virus triggers antiviral immune response in rainbow trout red blood cells, despite not being infective. F1000Res.

[60] Saraiva L.R., Ahuja G., Ivandic I., Syed A.S., Marioni J.C., Korsching S.I., Logan D.W. (2015). Molecular and neuronal homology between the olfactory systems of zebrafish and mouse. Sci. Rep..

[61] Casadei E., Salinas I. (2019). Comparative models for human nasal infections and immunity. Dev. Comp. Immunol..

[62] Richard M., van den Brand J.M.A., Bestebroer T.M., Lexmond P., de Meulder D., Fouchier R.A.M., Lowen A.C., Herfst S. (2020). Influenza A viruses are transmitted via the air from the nasal respiratory epithelium of ferrets. Nat. Commun..

[63] Singal C.M.S., Jaiswal P., Seth P. (2020). SARS-CoV-2, More than a Respiratory Virus: Its Potential Role in Neuropathogenesis. ACS Chem. Neurosci..

